# Medical Expert Testimony

**Published:** 1881-12

**Authors:** 


### MEDICAL EXPERT TESTIMONY.

The tendency of the times is toward bringing medical experts into disrepute. It must be admitted that the loss of confidence in such testimony is not without cause. Now and then instances are observed where the voice of the man, and of human nature, and not that of the physician, is uttered before the jury.

Questions of insanity have been the most potent cause of bringing the physician's opinion into disrepute with the courts and the public. There is here such an unavoidable range for opinions, that there must always be diverse and opposite views where a number of physicians can be reached by attorneys. The minds and views of physicians are as varying as the actions and eccentricities of lunatics. It is impossible that a well defined line of demarcation shall ever be drawn between sanity and insanity. We think it likely that there is no man but that some physician might be found to testify that his mind is unsound, under the manipulation of a shrewd lawyer. An attorney is, of course, allowed to select any physician as an expert whose views he has ascestained to be suited to the purpose at hand. It is charged that physicians in high places have received liberal fees for such testimony. Certainly an expert should receive a witness fee; but let it come from the proper source. It really seems that the courts, or some disinterested power, should have the selection of such experts as may be needed for the enlightenment of the jury. As it is, an energetic attorney may easily secure a medical man whose views are in accord with his own wishes. He can in all likelihood find one who is honest in such opinion; or, this failing, he may find one so unscrupulous as to manufacture opinions to order; and such an one, with the weight of his medical title attached. may impress a jury as well as an honest and more able physician.

Certain physicians seem to enjoy the notoriety or display of the witness stand. It seems to us a perverted taste. Such a fondness places them on about a par with the professional juryman. It seems to us, often, a degradation of the profession to have it paraded before an unappreciative jury by some shyster whose ignorance of medicine is exceeded only by his self-confidence. The physician must often submit to insults and insinuations in order that such an attorney may display his supposed shrewdness.

In short, to such an extent have the evils above mentioned been carried, that the opinions of medical experts are fast becoming a by-word and reproach. A high-minded and capable physician is reluctant to subject himself to the inconveniences of appearing as a witness, with the probability confronting him that his testimony may not exert its proper weight in securing right and justice. The remedy for all this is not easily pointed out; but we feel, as before hinted, that the court or the state, rather than the parties interested, should select the medical witnesses from whom is desired information on questions of medicine. This, under our system of government, is probably not a feasible plan. If not, then we must be content with efforts to raise still higher the honor and dignity of our profession.

---

The committee of the Medical Association of Georgia, on prize essays, consists of Drs. Eugene Foster, Henry F. Campbell, R. J. Nunn, W. A. Love and Robert Battey.

Dr. Foster is the chairman of the committee, and his address is Augusta, Ga.

